# Antimony, Arsenic and Chromium Speciation Studies in Biała Przemsza River (Upper Silesia, Poland) Water by HPLC-ICP-MS

**DOI:** 10.3390/ijerph120504739

**Published:** 2015-04-30

**Authors:** Magdalena Jabłońska-Czapla

**Affiliations:** Department of Waste Management and Environmental Analyses, Institute of Environmental Engineering, Polish Academy of Sciences, 34 Skłodowskiej-Curie St., 41-819 Zabrze, Poland; E-Mail: magdalena.jablonska-czapla@ipis.zabrze.pl; Tel.: +48-322-716-481 (ext. 126); Fax: +48-322-717-470

**Keywords:** HPLC-ICP-MS, speciation, arsenic, antimony, chromium, BCR, river, Biała Przemsza, heavy metals, metalloids

## Abstract

In this paper the total concentration of As, Cr, Sb, pH and the red-ox potential of water and sediment samples of the Biała Przemsza River were determined. The arsenic (AB, MMA, DMA, As(III), As(V)), chromium (Cr(III), Cr(VI)) and antimony (Sb(III), Sb(V)) forms were studied by HPLC-ICP-MS. Ions were successfully separated on Hamilton PRP-X100: (AB, MMA, DMA, As(III), As(V)), Dionex Ion Pac AS-7 (Sb(III), Sb(V)) and Dionex IonPac AG7 columns: Cr(III), Cr(VI) with LOD 0.16 μg/L, 0.08 μg/L, 0.09 μg/L, 0.012 μg/L, 0.08 μg/L, 0.12 μg/L, 0.009 μg/L, 0.012 μg/L, 0.19 μg/L, 0.37 μg/L, respectively. The simplified BCR three-step sequential chemical extraction was performed on the bottom sediment samples. The samples were collected monthly, between April and December 2014, at five sampling points. Large contents of manganese, lead, cadmium and zinc were found in the Biała Przemsza River water. In December 2014, the lead content in the bottom sediment in Sławków was nearly 6000 mg/kg. In the river water, only the inorganic arsenic speciation forms were found. Sb(V), As(V) and Cr(III) were dominant. Studies have shown that arsenic, antimony and chromium were mainly bound to oxides, organic matter and sulphides in the bottom sediments.

## 1. Introduction

The 21st century is a time of new challenges in the analytical chemistry field, and thus in the environmental analysis. There is new information on the toxicological characteristics of elements and their forms of occurrence. It is also necessary to detect and determine lower and lower analyte concentrations, often in complex matrix samples. The occurrence of various chemical and physical forms of a given element is known as “speciation” (a term borrowed from biology). Determining such forms is defined as speciation analysis [1]. However, following the IUPAC Recommendation 2000 [2], the term of “speciation” is used to determine the analytical activity to identify chemical individuals and measure their distribution in particular samples or matrices. Chemical speciation can also be defined as the process of identification and determination of specific chemical individuals, which helps to thoroughly understand the availability and mobility of metals so that their chemical behaviour can be comprehended. Speciation defines chemical forms, whereas fractionation is the process of classifying analytes or analyte groups in a given sample in terms of their physicochemical characteristics. Chemical speciation is an important research subject in environmental protection [3] and toxicological and analytical research as the toxicity, availability and reactivity of the trace elements depends on the chemical forms in which they occur. Within speciation analysis, the following aspects can be differentiated: determining substances produced and emitted into the environment by people, and analyzing natural compounds formed due to biochemical transformations in living organisms or in the environment. The former group is interesting for the environmental analysis, whereas the latter one is mainly studied by biochemists and eco-toxicologists.

Elements such as arsenic, antimony and chromium are the most interesting for toxicologists and analysts [4]. Depending on their oxidation states, they differ in characteristics. Antimony is common in the natural environment. It comes from natural processes and anthropogenic activity. Over the years, human activity has caused a large increase in the antimony concentration in the environment due to its use in the car industry (e.g., as an additive in the car tyre vulcanization). Antimony is similar to arsenic and bismuth in its geochemical behavior. Arsenic is a toxic metalloid that is common in the environment and various biological systems. The number of its speciation forms is constantly increasing due to the economic progress as the pollution caused by industry has not decreased in recent decades. On the contrary, the arsenic emissions from steel works, industry, animal waste and dust from fossil fuel combustion are rising at present. Arsenic is very mobile. Consequently, it occurs in all the environment elements. Its toxicity depends on the chemical form. Importantly, its inorganic speciation forms are 100 times more toxic than the organic ones. The contact with arsenic can cause various health effects, such as dermatological, inhalatory, cardiologic, genetic, genotoxic or mutagenic lesions [5]. The great interest in the Cr(III) and Cr(VI) forms is related to their different toxicological characteristics. Small amounts of chromium compounds are present in all living organisms in which Cr(III) plays an important role in the metabolic processes. It is a component of some enzymes and stimulates other organism activities. On the other hand, the Cr(VI) compounds are a serious threat for the environment due to their mutagenic and genotoxic characteristics. They are readily soluble, mobile and more bioavailable than Cr(III) compounds [6,7,8].

It turns out that the element form, rather than its total content, determines the influence the element has on living organisms. For that reason, the research into the toxic forms, such as As(III) and As(V), Sb(III), or Cr(VI), is so important. As those ions can cause many diseases/conditions, investigating their genotoxicity or metabolizing processes of these ions in living organisms has become popular. It is necessary to mention metabolomics here, which is a new science investigating all the metabolites present in organisms, tissues or cells. Analysing analytes at low concentration levels, particularly in complex matrix samples, requires more complex and sophisticated analytical methods and techniques. The latest trends in this field concern the so-called hyphenated methods (such as high performance liquid chromatography-inductively coupled plasma-mass spectrometry—HPLC-ICP-MS), in which the separation and detection methods are coupled [9,10,11].

Due to the long-term neglect, Upper Silesia is still “an environmental bomb”, and its rivers are a potential pollution source for other Polish regions. The Upper Silesian Rivers are among the most polluted ones in Poland. As they are tributaries to the main rivers (the Vistula and the Oder), they spread pollutants over the entire country area. Moreover, these rivers pose a serious threat for the inhabitants during floods that are often severe and cause permanent pollution of the flooded areas. The Biała Przemsza River is the last river in the Upper Silesian urban area which still maintains its natural character despite centuries of mining exploitation in its catchment area [12,13,14] leading to irreversible changes in the water environment. Nevertheless, the natural area in the river vicinity has been preserved and constitutes one of the most valuable areas in Silesia. The mining and metallurgic industries of the lead-zinc ores have developed in the Biała Przemsza catchment area and have had a strong influence on the surface water and bottom sediment condition.

As one of the stages of the MoSpeSil research project (Small Grant Scheme 2012; the Polish-Norwegian Research Programme) conducted in the Polish-Norwegian cooperation in 2013–2015, this study presents the research results for total contents of metals/metalloids using ICP-MS and arsenic, antimony and chromium speciation forms in the Biała Przemsza River water determined with HPLC-ICP-MS. Bottom sediments were extracted with the simplified BCR three-step sequential chemical extraction [15], and microwave digested, and then determined by ICP-MS. The research objective was to determine the mobility and seasonal changes of the arsenic, antimony and chromium speciation forms in the water and bottom sediments of highly polluted Upper Silesia river ecosystems using the Biała Przemsza River example.

## 2. Materials and Methods

### 2.1. Researched Area

The Biała Przemsza River flows through the Lesser Poland and Silesian Voivodships. It begins its course in the central part of the Polish Jura, *i.e.*, in the Olkuska Upland. It flows through the unique (on a European scale) Błędowska Desert and divides it into two parts. Close to Dąbrowa Górnicza, it mainly flows through marshes and swamps. These places are among the most wild and inaccessible ones. Despite the decrease in the water flow (typical for lowland waters), it has become a mountain river. The neighboring areas (forests, swamps) are inhabited by water organisms typical for the mountain ecosystems. The river character changes close to the point where it embraces its largest tributary, *i.e.*, the left-bank Biała River. The Biała Przemsza Valley visibly expands and becomes deeper. Below the Biała River mouth, the Biała Przemsza River changes radically. At present, the Biała River does not have its own natural sources, or they are only seasonal. The vast majority of the Biała River water is the mine water from the Pomorzany lead-zinc ore, discharged through the Dąbrówka canal. The remaining part comes from the wastewater treatment plant in Olkusz and small tributaries (a number of water fluxes on the left river side). The Biała River heavily pollutes the Biała Przemsza River, particularly in terms of heavy metal contents and suspension. In Okradzionów, the Biała Przemsza River radically changes its direction to the southern one. It starts meandering in Sławków and it gradually changes its direction to south-western one. Finally, it flows west. It flows through a thick and inaccessible forest, far away from the urbanized areas. There is another water tributary in the Burki residential area vicinity—the Sztoła River, which comes from the lead and zinc ore mine. In Mysłowice, the Biała Przemsza River flows into the Przemsza River (a left-bank tributary of the Vistula).

### 2.2. Sampling

**Figure 1:**
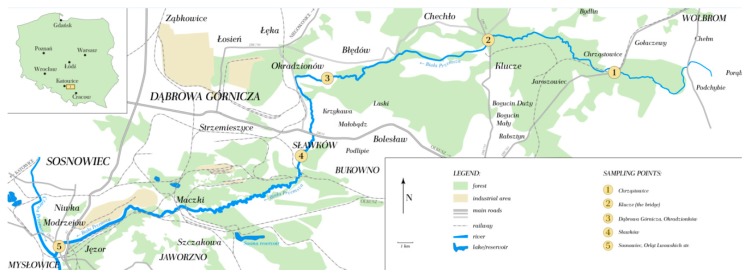
Location of the sampling points along the Biała Przemsza River.

The water and bottom sediment samples were collected monthly (April-December 2014) at five sampling points (BP1-BP5) shown in Figure 1. Directly after sampling *in situ*, the basic physicochemical parameters (pH, Eh, conductivity and temperature) were determined.

### 2.3. Sample Preparation

Directly after being transported into the laboratory, the water samples to be determined with ICP-MS were acidified with spectral grade nitric acid (V). Afterwards, they were filtered through a 0.22 µm PES syringe filter. The water samples for antimony speciation analysis were preserved with Na_2_EDTA. All the samples were kept at −22 °C (for no longer than a month). Finally, the samples were analyzed with the ICP-MS spectrometer to determine total analyte contents. After being averaged, the bottom sediment samples were air-dried, sieved through a sieve (2-mm mesh) and ground in a mortar. Next, they were mineralized in the Mars X microwave mineralizer (CEM Corporation, Matthews, NC, USA). The bottom sediment mineralization was optimized with the certified reference material (CRM) NCS DC 733309. The most effective mineralization was obtained when a mixture of 5 mL of HNO_3_ 65% (Merck, Darmstadt, Germany), 3 mL of 40% HF (Merck) and 2 mL of H_2_O_2_ (Merck) were used. The total concentration of metals and metalloids were determined in the obtained digests with ICP-MS spectrometer. The sieve analysis was performed in accordance with the Polish standard [16].

### 2.4. Research Methodology

The Elan 6100 DRC-e ICP-MS spectrometer (Perkin Elmer, Waltham, MA, USA) was used for quantitative analyses of the total arsenic, antimony and chromium concentrations in the samples of water, digests, and bottom sediment extracts. The device was equipped with a standard ICP quartz torch, cross-flow nebulizer, and nickel cones. The samples and standards were fed with a peristaltic pump. The spectrometer was optimized daily with 10 µg/Lsolution (Mg, Cu, Rh, Cd, In, Ba, Ce, Pb, U) in 1% HNO_3_ Elan 6100 Setup Solution (Perkin-Elmer). The ^53^Cr, ^75^As and ^123^Sb concentrations were measured with the Rh as internal standard. The operating parameters for the ICP-MS spectrometer are given in Table 1. The simplified BCR three-step sequential chemical extraction [16] helped to determine the forms of arsenic, antimony and chromium in the bottom sediments and the way in which they were bound. The conditions under which the sequential extraction was performed are given in Table 2. The concentration speciation of chromium (Cr(III), Cr(VI)), arsenic (As(III), As(V), AsB, MMA, DMA)) and antimony (Sb(III), Sb(V)) forms were determined by HPLC-ICP-MS. The optimized separation and determination parameters for the arsenic, antimony and chromium forms are given in Table 3. The method validation parameters are shown in Table 4. The following substances were used for analyses: ultrapure ammonium nitrate (Merck, Darmstadt, Germany), ultrapure potassium dichromate (Merck), 1000 mg/L Cr(III) standard solution (Merck), sodium dihydrogen arsenate heptahydrate (Sigma-Aldrich, St Louis, MO, USA), sodium arsenite (Sigma-Aldrich), disodium methyl arsenate (Supelco, Bellefonte, PA, USA), arsenobetaine (Sigma-Aldrich, St Louis, MO, USA), dimethylarsinic acid (Supelco), potassium hexahydroantimonate(V) (Aldrich), phthalic acid (POCH, Gliwice, Poland). The calibration solutions were prepared each time through diluting suitable standard solutions on an analytical balance. The multi-element standards no. XXI and VI (Merck) were used when determining total arsenic, antimony and chromium with ICP-MS. Solutions made from salts were employed for calibration during quantitative determinations of arsenic, antimony and chromium speciation forms. All solutions and standards were prepared with the Milli-Q-Gradient ultrapure deionized water (Millipore, Merck Darmstadt, Germany), whose electrolytic conductivity was <0.05 µS/cm.

**Table 1:** ICP-MS spectrometer operating parameters.

ICP-MS Parameter	Settings
Generator power RF (W)	1125
Plasma gas flow (l/min)	15
Nebulizer gas flow (l/min)	0.76–0.82
Auxiliary gas flow (l/min)	1.15–1.16
Nebulizer	cross
Torch	quartz
Scanning mode	Peak hopping
Dwell time (ms)	250
Sweeps/Reading	1
Number of replicates	830

**Table 2:** BCR sequential chemical extraction procedure.

Fraction	The Extraction Solution	Associated With
F1 Exchangeable	Acetic acid: 0.11 M CH_3_COOH, pH = 2.85, shaking for 16 h, the ratio of solid / solution 1:40 Room temperature	Loosely adsorbed cations and anions on sediments and carbonates and very reactive oxy-hydroxides
F2 Reducible	Hydroxyl ammonium chloride: 0.1 M NH_2_OH^.^HCl, pH = 2.0 shaking for 16 h, the ratio of solid / solution 1:40 Temperature 85 ± 2 °C	Iron/manganese oxides (mostly amorphous or poorly crystallized)
F3 Oxidisable	Perhydrol: H_2_O_2_ 8.8 M, CH_3_COONH_4_ 1.0 M pH = 2.0	Organic substance and sulphides
Residual	Aqua regia: 3HCl + HNO_3_	Non silicate bound metals

**Table 3:** Separation parameters.

Parameter	Value
***Chromium***	
Column	Ion Pac AG-7; 50 mm × 4 mm, 10 µm
Temperature	35 °C
Mobile phase	A: 0.1M NH_4_NO_3_ pH = 4; B: 0.8M HNO_3_
Elution program	0–0.5 min 100% A, 1.5–3.5 min 100% B rinsing 3.5–5.0 min. 100% A
Flow rate during the analysis (mL/min)	1.7
Flow rate during the rinsing (mL/min)	2.0
Volume of sample (µL)	170
***Antimony***	
Column	Ion Pac AS-7; 200 mm × 4 mm, 10 µm
Temperature	35 °C
Mobile phase	1mM phthalic acid, 10mM EDTANa_2_; pH = 4.5
Elution time	3 min
Flow rate during the analysis (mL/min)	1.2
Volume of sample (µL)	80
***Arsenic***	
Column	Hamilton PRP-X100; 100 mm x 4 mm, 10 µm
Temperature	30 °C
Mobile phase	A: 20mM NH_4_NO_3_ pH = 8.7 B: 60mM NH_4_NO_3_ pH = 8.7
Elution time	0–2.0 min. 100% A 2.0–3.0 min from 100% A to 100% B 3.0–6.5 min 100% B rinsing 6.5–9.5 min 100% A
Flow rate during the analysis (mL/min)	1.1
Volume of sample (µL)	100

**Table 4:** Validation parameters.

Analyte	Limit of detection (µg/L)	Recovery (%)	Relative Standard Deviation of Repeatability (%)	Uncertainty (%)
Cr (total)	0.013	116	5.9	23
Cr(III)	0.19	99	2.0	14
Cr(VI)	0.37	102	2.3	11
Sb (total)	0.005	106	3.2	12
Sb(III)	0.009	105	1.9	8
Sb(V)	0.012	101	2.4	8
As (total)	0.096	88	4.8	29
As(III)	0.08	96	2.9	12
As(V)	0.12	104	2.4	11
AB	0.16	93	3.7	17
MMA	0.08	95	3.1	12
DMA	0.09	95	2.7	11

## 3. Results

**Figure 2:**
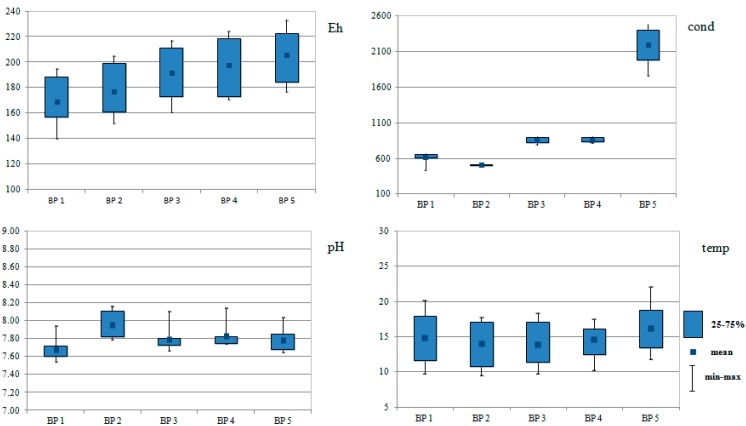
Box plots of pH, conductivity (µS) (cond), temperature (°C) (temp) and redox potential (Eh) data for Biała Przemsza river monitoring (five sampling points BP1-BP5, samples taken from April to December 2014 Biała Przemsza River water).

The measurements of the basic physicochemical parameters demonstrated that the lowest pH was found in the Biała Przemsza River water samples in Chrząstowice (BP1), whereas the highest pH values were measured in Klucze (BP2). The pH value decreased at the following water sampling points (BP3-BP5; downward direction of the river course). Similarly to pH, the redox potential had the lowest value at BP1. It gradually rose along the river course. The highest conductivity value was observed at BP5 in Sosnowiec, where the highest ion concentrations were found. Figure 2 shows the variation of physical and chemical parameters of Biała Przemsza water samples.

**Figure 3:**
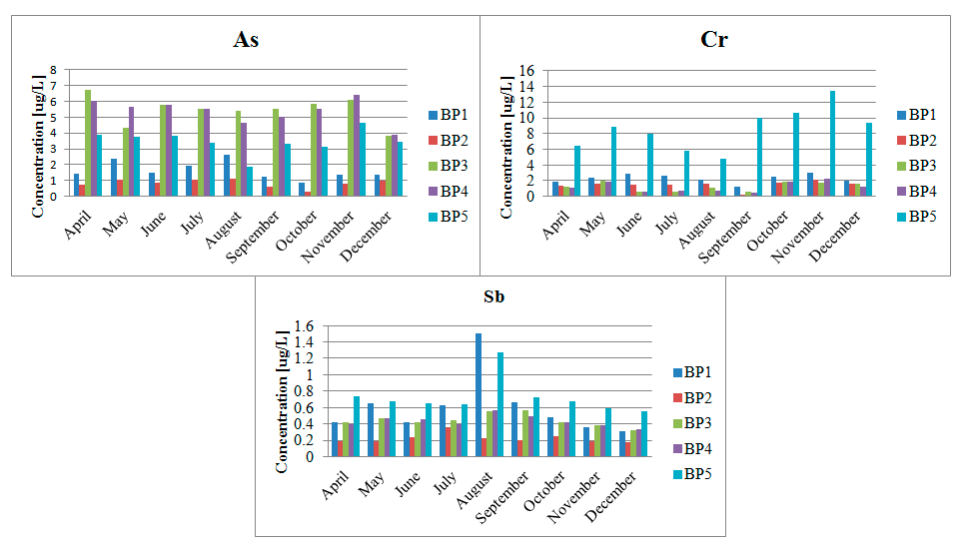
Total contents of arsenic, antimony and chromium in the Biała Przemsza River water; sampling points: BP1—Chrząstowice; BP2—Klucze Osada; BP3—Dąbrowa Górnicza Okradzionów; BP4—Sławków; BP5—Sosnowiec.

**Figure 4:**
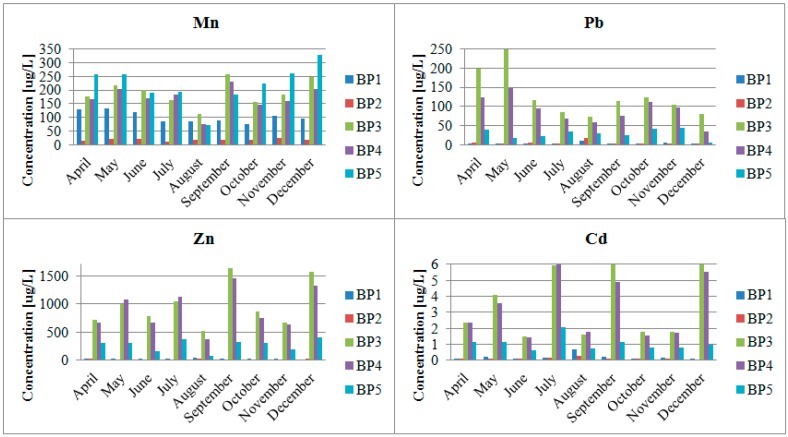
Total contents of manganese, lead, cadmium and zinc in the Biała Przemsza River water; sampling points: BP1—Chrząstowice; BP2—Klucze Osada; BP3—Dąbrowa Górnicza Okradzionów; BP4—Sławków; BP5—Sosnowiec.

As shown in Figure 3, the highest chromium concentration was observed at the BP5 sampling point. It was nearly 14 µg/L. The highest arsenic concentrations in the Biała Przemsza River water were measured in Sławków and Dąbrowa Górnicza Okradzionów. In the case of antimony, the highest concentrations were observed at BP1 and BP5. These analyte contents were low enough to assign the Biała Przemsza River water to Class I [17]. Unfortunately, the low trace concentrations of arsenic, antimony and chromium were accompanied by high contents of manganese, lead, cadmium or zinc (Figure 4). The Biała Przemsza River flows through areas rich in the zinc ores. What is more, the highly-developed mining and metallurgic industries of the lead-zinc ores are present in its catchment area. They strongly influence the condition of the surface water and bottom sediments.

**Figure 5:**
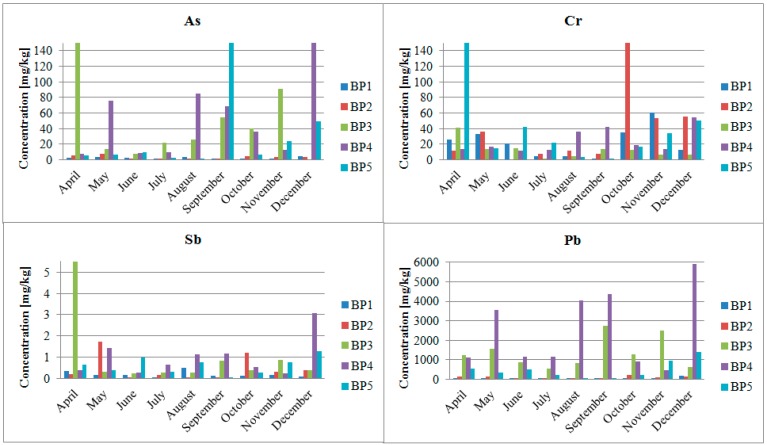
Total contents of arsenic, antimony, chromium and lead in the Biała Przemsza River bottom sediments; sampling points: BP1—Chrząstowice; BP2—Klucze Osada; BP3—Dąbrowa Górnicza Okradzionów; BP4—Sławków; BP5—Sosnowiec.

Figure 5 presents total contents of arsenic, antimony, chromium and lead in the Biała Przemsza bottom sediments. The highest arsenic and antimony contents were observed at BP3 and BP4 in April. In the same month, very high chromium content was found at BP4 (Sławków). The bottom sediments were highly polluted with lead. The highest concentrations were measured in Sławków (nearly 6000 mg/kg in December) and Dąbrowa Górnicza.

**Figure 6:**
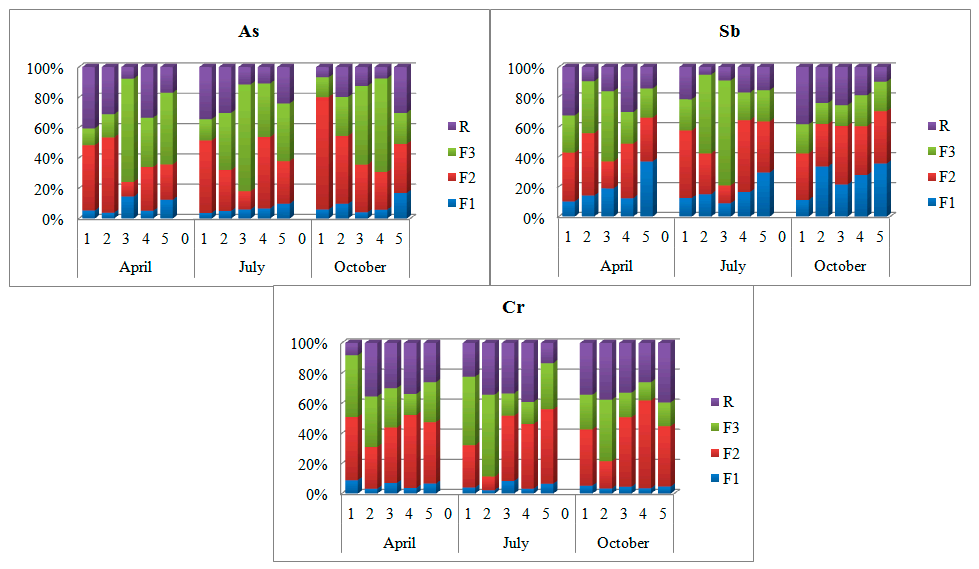
Simplified BCR three-step sequential extraction [15]. F1—ion-exchange and carbonate fraction; F2—oxide fraction; F3—organic and sulphide fraction; R—residual; sampling points: BP1—Chrząstowice; BP2—Klucze Osada; BP3—Dąbrowa Górnicza Okradzionów; BP4—Sławków; BP5—Sosnowiec.

The results of a simplified three-step sequential chemical extraction showed Figure 6. The sieve analysis (Table 5) showed that the Biała Przemsza River bottom sediments mainly contained the particle fractions of 0.5–0.2 mm, *i.e.*, medium and fine sand.

**Figure 7:**
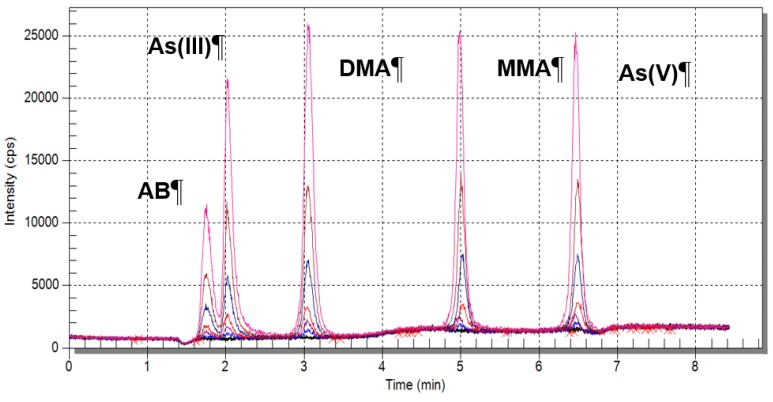
Chromatograms of the arsenic speciation form standard solutions with concentrations of 20 µg/L, 10 µg/L, 5 µg/L, 2 µg/L, 1 µg/L, 0.1 µg/L.

The application of HPLC-ICP-MS helped determine five organic and inorganic arsenic speciation forms, two antimony speciation forms and two chromium speciation forms. Figure 7 presents chromatograms of the standard solutions used for calibration. The concentration of arsenic, antimony and chromium speciation forms were determined with low limits of detection (Table 4) [18].

**Figure 8:**
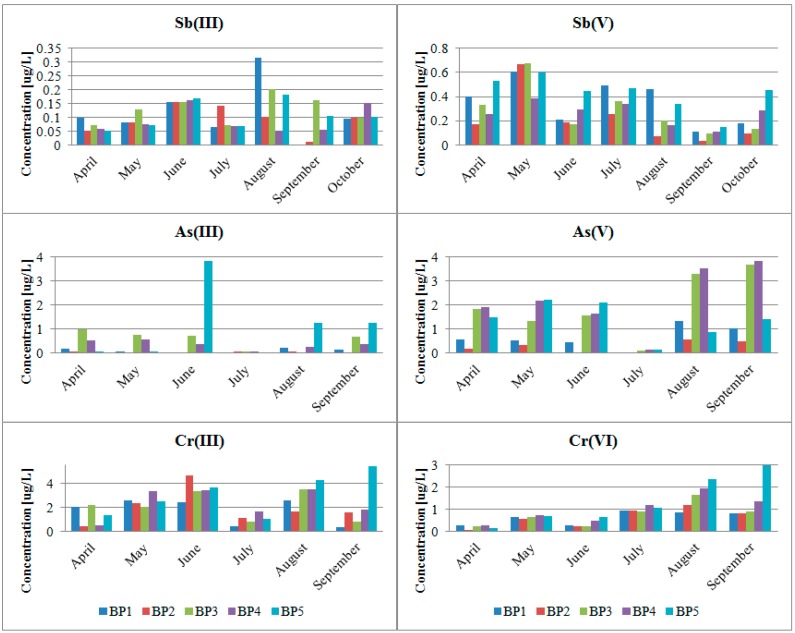
Concentrations of Sb(III)/Sb(V), As(III)/As(V) and Cr(III)/Cr(V) in the Biała Przemsza River water; sampling points: BP1—Chrząstowice; BP2—Klucze Osada; BP3—Dąbrowa Górnicza Okradzionów; BP4—Sławków; BP5—Sosnowiec.

Table 6 presents the minimum, maximum and median contents of the arsenic and chromium speciation forms in the Biała Przemsza River water. Figure 8 shows concentrations of Sb(III)/Sb(V), As(III)/As(V) and Cr(III)/Cr(V) in the Biała Przemsza River water.

**Table 5:** The sieve analysis of the Biała Przemsza River bottom sediments; sampling points: BP1—Chrząstowice; BP2—Klucze Osada; BP3—Dąbrowa Górnicza Okradzionów; BP4—Sławków; BP5—Sosnowiec.

Date of Sampling	Sampling Point	>2.0 mm	2.0–1.0 mm	1.0–0.5 mm	0.5–0.2 mm	0.2–0.1 mm	<0.1 mm	Unit
April	BP1	0.16	0.22	15.79	77.85	3.64	0.17	%
	BP2	0.29	0.17	15.54	73.77	10.06	0.18	%
	BP3	5.51	11.94	12.47	31.89	25.85	12.34	%
	BP4	0.00	0.01	2.27	88.87	8.78	0.08	%
	BP5	0.07	0.23	2.00	80.85	15.44	1.41	%
July	BP1	0.16	0.22	15.79	77.85	3.64	0.17	%
	BP2	2.43	2.45	6.32	63.08	23.17	3.68	%
	BP3	1.30	0.75	1.02	36.36	34.93	26.94	%
	BP4	0.00	0.00	0.39	78.43	20.22	0.96	%
	BP5	1.26	1.48	8.77	80.18	7.55	0.76	%
October	BP1	0.07	0.11	9.25	84.21	6.09	0.28	%
	BP2	11.97	3.19	5.74	38.08	31.42	9.60	%
	BP3	0.08	0.00	0.92	59.91	33.03	6.06	%
	BP4	0.18	0.11	0.31	32.10	30.28	37.02	%
	BP5	0.52	0.76	4.46	82.38	10.82	1.05	%

**Table 6:** Minimum, maximum and median contents of the arsenic and chromium speciation forms in the Biała Przemsza River water; sampling points: BP1—Chrząstowice; BP2—Klucze Osada; BP3—Dąbrowa Górnicza Okradzionów; BP4—Sławków; BP5—Sosnowiec.

(µg/L) N = 30		BP1	BP2	BP3	BP4	BP5
As(III)	minimum	<0.08	<0.08	<0.08	0.26	<0.08
	maximum	0.21	0.08	0.99	0.56	3.83
	median	0.11	0.08	0.72	0.39	1.24
As(V)	minimum	<0.12	<0.12	0.13	0.16	0.17
	maximum	1.35	0.56	3.67	3.83	2.22
	median	0.55	0.26	1.71	2.05	1.45
Cr(VI)	minimum	<0.37	<0.37	<0.37	<0.37	<0.37
	maximum	0.95	0.97	0.93	1.19	1.07
	median	<0.37	<0.37	<0.37	0.51	0.67
Cr(III)	minimum	0.44	0.41	0.86	0.51	1.07
	maximum	2.59	4.63	3.33	3.41	3.65
	median	2.24	1.74	2.13	2.51	1.92

## 4. Discussion

### 4.1. Impact of the Physicochemical Parameters on the Concentration of Metal (Loid)s and their Forms in Biała Przemsza River Waters

The influence of physicochemical parameters on the variability of an element forms concentrations is complex, and depends on the pH, conductivity, redox potential and temperature. The pH decrease led to the dissolution of carbonates and hydroxides, and the metal ions were displaced with the hydrogen ions. The increase in the salt content in water caused the shift of the sorption isotherm into the higher pH direction or led to the formation of the soluble metal-chloride ion complexes. The change in the redox potential caused the change in the metal binding forms in the solid phase and the pH drop, which increased the metal mobility. The pH value decreased downward direction of the river course. Similarly to pH, the redox potential had the lowest value at first sampling point.

Thus, the increase in the concentration of metal(lod)s and their forms in the Biała Przemsza River waters is probably also assisted by occurring at the same time increase the redox potential and pH decrease along the river. The effect of specific physicochemical conditions and a general increase in pollution in the last water sampling point (in Sosnowiec, just before Biała Przemsza River estuary), is the highest observed concentration of chromium and antimony in the Biała Przemsza waters.

The highest conductivity value observed at last sampling points, was concerned with the highest ion concentrations (mainly chlorides and sulphates). Such a finding confirms the high salt contents in the supplying mine water. Mine water discharges in the Upper Silesian Rivers are a big problem not only in this area [19].

### 4.2. Impact of the Pb and Zn Ore Mining Industry on the Pollution of the Biała Przemsza River

The aim of the study was to investigate the content of arsenic, antimony and chromium and their forms in water and bottom sediments of the White Przemsza. However, a very important parameter is also during the study holistic approach to the subject, containing also a very rich martix of samples taken from the Biała Przemsza River. The largest industrial facility in the Biała Przemsza River catchment area is ZGH “Bolesław” in the Olkusz area, which is known for its lead-zinc ore exploitation. That is why the particularly high contents of cadmium, lead and zinc were found in Dąbrowa Górnicza (Okradzionów) and Sławków. High contents of manganese were accompanied by high contents of cadmium and zinc in September (Figure 4). The lowest manganese concentration was found in Klucze. A slightly higher one was observed at BP1 in Chrząstowice (approximate 100 µg/L). The Biała Przemsza River demonstrated mountain character at these two sampling points. Its water was clear and the current was rapid. Over the whole year, the manganese concentration demonstrated a downward trend between April and August. Afterwards, it increased until December 2014, when it reached 326 µg/L at BP5 (Sosnowiec). Due to the total manganese content in the Biała Przemsza River water, it can be classified within Classes II and III of surface water purity. In terms of the manganese content, the water quality was very good (Class I) [20] only at BP2 (Klucze). Polich-Latawiec *et al.* [12] showed that the mean manganese content in the Sztoła River water (the left-bank Biała Przemsza River tributary) was 30–40 µg/L. The Sztoła River which additionally pollutes the researched river with manganese, lead, cadmium and zinc flows into the Biała Przemsza River at Sławków. The Biała River and Sztoła River supplied the mine water from the Pomorzany lead-zinc ore mine, wastewater from the ZGH “Bolesław” in Bukowno and wastewater treatment plant in Olkusz. Below their mouth, the Biała Przemsza River water became seriously polluted with heavy metals and other elements. Importantly, the manganese content largely exceeded 1 mg/L in May, July, September and December. According to the regulations [17], the zinc content in the river water should also be lower than 1 mg/L. In its lower part, the mean contents of manganese, lead and zinc in the Biała Przemsza River water were 160 µg/L, 35 µg/L and 540 µg/L, respectively [21].

The manganese content was 18–89 µg/L [22] in Chinese rivers such as the TongYu River, Taidong River or Mangshe River. In comparison, the manganese content in the Biała Przemsza River was relatively high (100–350 µg/L), except for the low manganese content at BP2 in Klucze.

The zinc content varied. In Chrząstowice, the river water was Class I. On the other hand, it was Class III–IV in Dąbrowa Górnicza and Sławków [20]. The cadmium content was 0.11–6.67 µg/L. Consequently, the Biała Przemsza River water could be classified within Classes I–III for surface water purity [20]. Due to large lead contents (significantly exceeding 50 µg/L), the river water could not be assigned to any Class.

The highest contents of cadmium, lead and zinc were observed at BP3 and BP4. Such a situation was caused by the Biała Przemsza tributaries. The Biała River, which flows in the Biała Przemsza just ahead of BP3 in Okradzionów, does not have natural sources at present, or they appear seasonally. The majority of the Biała River water is constituted by the mine water from the Pomorzany lead-zinc ore mine, discharged through the Dąbrówka canal. The remaining part comes from the wastewater treatment plant in Olkusz and small tributaries (a number of water fluxes on the left river side). The Biała River heavily pollutes the Biała Przemsza River, particularly in terms of heavy metal contents and suspension.

### 4.3. Metal (Loid)s and their Forms in Biała Przemsza Bottom Sediments

#### 4.3.1. BCR Extraction Procedure

The results show that arsenic was mainly bound to the oxide and organic-sulphide fractions in the Biała Przemsza bottom sediments. At BP1 and BP2, arsenic was mainly bound to oxides and residual fractions in spring season. At BP2 (Klucze), arsenic occurred in the oxide form, except for summer when it was mainly bound to the organic fraction of the plant origin. At the remaining points, arsenic was bound to the oxide and organic-sulphide fractions. Small arsenic amounts were also bound to the ion-exchange fraction. In third sampling point, arsenic was mainly bound to the organic-sulphide fraction. According to other authors generally, arsenic and antimony are mainly bound to iron hydroxides in the river bottom sediments [23]. The bottom sediment research in the Biała Przemsza River confirmed this information. The highest arsenic concentration was observed in the oxide fraction, particularly at BP1 and BP2.

For antimony, there were seasonal changes in the ion-exchange fraction percentage. It increased during the year and the highest value was found at BP5. The antimony percentage was fairly averaged in particular fractions. The only exception was the sample collected at BP3 in July, where the antimony demonstrated the highest percentage in the organic-sulphide fraction as the bottom sediment was rich in the organic matter at that time. In comparison to arsenic and chromium, the proportion of antimony concentration in the ion exchange fractions was greatest.

Chromium at BP3-BP5 sampling points, was mainly bound to the oxide and residual fractions. At the first two sampling points chromium was also strongly associated with the organic-sulphide fractions.

The Biała Przemsza River is the last river in the Upper Silesian urban area which still maintains its natural character despite centuries of mining exploitation in its catchment area. The mining works have caused irreversible changes in the water environment. Nevertheless, the natural area in the river vicinity has been preserved and constitutes one of the most valuable areas in Silesia. The Biała Przemsza River bottom sediments are rich in lead, cadmium and zinc. They also contain significant amounts of the demobilized arsenic, antimony and chromium which are mainly bound to oxides, organic matter and sulphides. From the biological viewpoint, metals/metalloids bound to the ion-exchange fraction are the most harmful due to their bioavailability.

Due to the surface water self-purification processes, dissolved heavy metal forms are transported into the bottom sediments during sorption and other biochemical processes. Consequently, the water quality could be improved, but the metal concentrations in the bottom sediments increase. The heavy metal content in the bottom sediments is a good indicator for the water environment pollution level [24,25]. At present, it is very popular to calculate the heavy metal pollution index (HPI) [26]. The HPI represents the total water quality in respect to heavy metals.

There are many literature reports on the research into metal and metalloid contents in the river ecosystems [27,28,29,30]. The metal and metalloid speciation from bottom sediments mainly concerns various fractionation procedures, such as BCR [31], Tessier [32] and their modifications [33].

#### 4.3.2. Speciation Analysis Using HPLC-ICP-MS Technique

Only the inorganic arsenic forms were found in the Biała Przemsza River water. The content of the organic arsenic speciation forms (e.g., MMA, DMA and AB) was below the limit of quantification. The fact that there were only inorganic arsenic forms present proves that the Biała Przemsza environmental condition was quite good. The oxidised arsenic form dominated, particularly at BP1 and BP2. In Chrząstowice and Klucze, the Biała Przemsza had still the character of a clear mountain river. From Dąbrowa Górnicza Okradzionów on, the Biała Przemsza River became much more polluted, mainly by its tributaries. That is why, there were large contents of the reduced arsenic form in its water. The highest As(V) concentration was observed at BP3 and BP4 in August and September. Special situation was observed in June 2014, when reported an extremely high concentration of As(III) 3.83 µg/L in a water sample taken from the last sampling point (BP5). Concentration of reduced, ten times more toxic, arsenic form, almost double exceeded the concentration of the oxidized arsenic forms. The obtained results are supported with the total arsenic content in water (Figure 3).

The reduced chromium form dominated in the Biała Przemsza River water. The Cr(VI) content dominated only at BP1. It was comparable at the remaining points in Spring 2014. Such a situation was not random and could have been related to the favourable redox conditions at that time.

Results show that the oxidised antimony form dominated in the Biała Przemsza water. Its maximum value was measured in May 2014. Afterwards, it gradually decreased until September 2014. There was an increase in the reduced antimony form in August-September. The highest Sb(III) concentration (0.31 µg/L) was observed at BP1 in August.

There are not many environmental river studies discussing metal and metalloid speciation determined with hyphenated analysis methods. HPLC-ICP-MS was successfully used for studying the water of the Kamo-Ichinokawa river system in Japan [34]. The oxidation state of antimony in all the river samples determined with HPLC-ICP-MS was pentavalent, possibly dissolved as Sb(OH)_6_^−^. When Miyashita *et al.* [35] investigated the Hayakawa River (Japan), they found only the As(V) content. The organic speciation arsenic forms (e.g., MMA, DMA, AsB or arsenosugars) were only found in the researched fish and other aquatic living organisms. The dominant arsenic oxidation state in the river water was pentavalent. The chromium forms in research conducted in the Sacramento River (California, CA, USA) [36] demonstrated the presence of both speciation analyte forms. Similar results were obtained for the Biała Przemsza River. On average, the Cr(III) content was 2–3 times higher than the Cr(VI) one. Nonetheless, the Cr(VI) level was approx. 1 µg/L in the Biała Przemsza River water.

## 5. Conclusions

The application of HPLC-ICP-MS enabled speciation analysis of arsenic, antimony and chromium forms at the ultra-trace levels. The optimized method was selective and has a low detection limits. The repeatability, intermediate precision and accuracy allow for its use in trace analysis of environmental samples.

The obtained results confirm prior literature studies. Apart from a few exceptions, due to the contents of the toxic Cr(VI), Sb(III) or As(III) forms, the Biała Przemsza River can be classified as a river that does not pose environmental threat and significant ecological hazard. On the other hand, contents of cadmium, lead, manganese or zinc were also observed. Their presence was caused by well-developed mining and metallurgic industries in the Biała Przemsza catchment area. These industries influenced the condition of the surface water and bottom sediments. The largest industrial facility present in the Biała Przemsza catchment area is ZGH “Bolesław” (known for lead-zinc ore exploitation), located in the Olkusz area. Unfortunately, due to its activity, large amounts of elements were found in the Biała Przemsza River water and bottom sediments.
